# The English-Only Nature of Vaccine Deservedness and Harms of Racialized Administrative Burdens

**DOI:** 10.1007/s40615-025-02507-5

**Published:** 2025-06-27

**Authors:** Sayil Camacho, Carolyn J. Heinrich, Montrell Sanders, Mary-Margaret McGowan

**Affiliations:** 1 Latina Futures 2050 Lab, University of California, Los Angeles (UCLA), 193 Haines Hall, Los Angeles, CA 90095-1544 USA; 2 Peabody College of Education and Human Development, Vanderbilt University, 230 Appleton Place, Nashville, TN 37203 USA; 3 Department of Public Policy and Administration, Rutgers University-Camden, 401 Cooper Street, Camden, NJ 08102 USA

**Keywords:** Limited English Proficient (LEP) populations, Healthcare equity, Racialization and discrimination, Structural humility

## Abstract

Despite extensive efforts to facilitate COVID-19 vaccine access and achieve a 70% vaccination rate, race-based disparities and barriers to vaccine access continue to persist in the United States. To investigate discrimination among Limited-English Proficient (LEP) populations, we conducted a paired audit test field experiment. Testers with identical gender and immigration status, but differing in racial and language profiles, inquired about vaccine access in all 50 states through the Department of Health and Major Vaccine Sites, using phone, email, and web-based chat communication. The results show that Spanish-speaking testers faced additional systemic barriers, were more likely to encounter overt racialized and judgmental language, and experienced heightened scrutiny regarding their identity. These findings highlight both macro-level communication infrastructure and micro-level interactions that contribute to ongoing disparities in healthcare access.

## Introduction

Amid a global pandemic and the re-energized civil rights movement, the U.S. Department of Homeland Security (DHS) released a statement supporting equal access to the COVID-19 Vaccine and Vaccine Distribution Sites [1, 2]. Proclaiming a moral and public health imperative, DHS established its support for all people, regardless of residency status, to receive the COVID-19 vaccine per local distribution guidelines. To advance these efforts, the Centers for Disease and Control (CDC) Vaccine Task Force affirmed sufficient vaccine supply for open eligibility [3], implying that supply issues should not preclude states from offering the vaccine to non-residents [4–7]. The Federal Emergency Management Agency (FEMA) Office of Equal Rights also instituted a Civil Rights Advisory Group to ensure nationwide equity in vaccine access and distribution [8], recognizing that historically excluded populations were simultaneously more likely to be exposed and contract COVID-19, have lesser access to vital healthcare services, and be overrepresented among the essential and low-wage workforce [9–11].

Although the National COVID-19 Preparedness Plan coordinated a whole-of-government effort to surpass the 70 percent threshold for population immunity, vaccine uptake remained low among historically excluded populations [12–14]. Increased exposure to COVID-19 and disparate access to healthcare is not a race-neutral experience [15]. For example, as State Departments of Health (SDH) and major vaccination sites (MVS) carried out the National COVID-19 Preparedness Plan, emerging studies suggested that the lowest vaccine rates were among Spanish-speaking Latinx/e people [16], and vaccine equity studies confirmed that undocumented immigrants experienced additional barriers in accessing the COVID-19 vaccine [17, 18]. Simultaneously, an estimated 69 percent of immigrants and 74 percent of undocumented people were essential workers [19].

The especially low vaccine rates among Spanish-speaking Latinx/e individuals and undocumented immigrants reflect the structurally marginalized position these populations occupy in U.S. society. Shaped by socially constructed notions of race, ethnicity, and belonging [20] markers such as perceived national origin, language spoken [21], and social class [22–24] contribute to how these populations are racialized within dominant societal frameworks. For instance, research shows that identification as Latinx/e and/or Spanish-speaking is often conflated with assumptions of undocumented status, even though the majority of Latinxs are U.S.-born (68%), and a minority of the Latinx/e population (12.2%) is undocumented [25, 26]. Language, like phenotype or surname, plays a central role in the racialization of Latinx/e individuals and contributes to the social and psychological process construction of ethnic identity [27]. These compounding dynamics illustrate how Latinx/e populations are burdened with contemporary notions of “illegality” rooted in racialized assumptions about language, origin, and class.

To interrogate this phenomenon, we conducted a field experiment to examine how racial and language differences influenced vaccine access in all 50 states (DHS and MVS). Testers—an English-speaking White, an English-speaking Latinx/e, and a Spanish-speaking Latinx, each with racially and ethnically corresponding surnames—followed an identical procedure to inquire about vaccine access via phone, email, and chat communication. The objective was to evaluate how states communicated vaccination requirements and whether that varied by perceived race and language of the tester and across states. Our empirical analyses confirmed that states varied considerably in how clearly officials communicated vaccination requirements and procedures, and in what they asked of individuals before providing the opportunity to receive the COVID-19 vaccine. Using the lens of the *Structural Humility* framework, a healthcare approach that facilitates the identification of structural systems of power that marginalize historically excluded populations [28–30], we undertook a qualitative data analysis to assess whether variation in those interactions were associated with the racialization of the tester.

This study informs the development of a more structurally competent healthcare system by identifying root causes of inequality in vaccine access and offering policy recommendations to address these barriers—particularly for Latinx/e Spanish speakers who constitute nearly two-thirds (62%) of the Limited English Proficient (LEP) population [31]. Our findings highlight inequitable interactions that reinforce healthcare disparities and point to structural changes necessary to promote healthcare equity.

## Literature Review

### The Racialization of Language and COVID-19 Vaccine Access

Research has identified four major barriers to equitable healthcare access for LEP patients: (1) communication and health literacy, (2) relationships with healthcare professionals, (3) discrimination, and (4) cultural safety [32]. LEP experiences in healthcare are not race-neutral. Historically excluded populations face compounded marginalization [33, 34], and the intersections of historically excluded identities perpetuate added forms of marginalization within and outside of healthcare experiences [33, 34]. Relatedly, studies of administrative burden find that historically underserved and disadvantaged groups struggle the most in overcoming bureaucratic barriers to benefits and services [35–37], reinforcing racialized inequalities in accessing benefits like vaccines [38].

The majority of LEP individuals are Latinx/e Spanish speakers (63%) and are disproportionately concentrated in four states—California (25%), Texas (14%), Florida (10%), and New York (9%)—which together constitute 60% of the LEP populations [31]. LEP status among the Latinx/e Spanish-speaking population has been consistently linked to lower healthcare access and quality of care [39], with linguistic isolation contributing to reduced quality of life, delayed healthcare access, and lower vaccination rates [40, 41]. Among Spanish-speaking LEP populations, added vaccine access barriers exacerbate disparities in preventative care, including vaccination rates [42, 43]. The COVID-19 pandemic magnified these vulnerabilities, especially for newly arrived immigrants [44, 45] who experienced higher hospitalization and mortality rates and were less likely to receive the vaccine [46].

Among LEP immigrant populations, particularly Spanish-speaking Mexican Americans, misinformation, insecurity, and distrust of institutions remain key barriers to vaccine uptake [47]. This aligns with past findings on LEP patient experiences, where language barriers, healthcare discrimination, and reliance on family members for translation contributed to inadequate care [32]. In this context, Latinx/e Spanish-Speaking LEP populations have reported increased stress within healthcare settings due to limited translation resources and services, as well as feeling vulnerable and disempowered [32, 48, 49].

### Structural Humility to Address Disparate Healthcare Inequities

A growing body of scholarship has developed culturally competent communication strategies to address healthcare inequities faced by LEP populations [50–52]. In addition to complying with federal and state language access guidance standards, such as the National Standard for Culturally and Linguistically Appropriate Services (CLAS), culturally competent approaches include the following: (1) identifying language barriers beyond basic translation services; (2) developing accessible health literacy resources using popular education techniques; (3) disseminating resources across communication platforms (i.e., text, audio, and visual); (4) providing ongoing cultural competency training for all healthcare professionals and staff to ensure available cultural brokers; and (5) launching community-based information campaigns available in physical proximity to historically excluded populations [53–57].

In regard to vaccine access, medical literature recommends strategies tailored to vaccine hesitancy levels and community-specific needs. These include simplifying vaccine take-up, providing reminders and incentives through trusted networks, and delivering targeted educational messaging [58–60]. We used this literature to guide the evaluation of communications between the audit testers and SDH and MVS personnel during our field experiment. Broadly, this research affirms the promise of culturally competent communication strategies, systemwide change, and epistemic humility as pathways for better understanding and addressing healthcare inequities [61].

While a necessary foundation for equitable healthcare, cultural competency is not sufficient on its own. The structural humility framework we employ goes beyond interpersonal awareness to interrogate the institutional and systemic foundations of healthcare inequity. Structural humility emphasizes the need to (a) recognize power asymmetries built into healthcare delivery, (b) shift burdens away from historically excluded populations, and (c) facilitate critical self-reflection among healthcare institutions and providers about their role in reinforcing structural barriers [29, 30, 62, 63]. This framework shaped both our analytical approach and interpretation of audit tester interactions, allowing us to assess not only what was said, but how communication systems obstructed equitable vaccine information and access.

Designed to augment culturally competent forms of health-care approaches [29, 64–66], structural humility posits that racial assumptions, tensions, and biases manifest due to the centrality of racism within the healthcare system and discipline. The racism espoused by both the healthcare system and discipline is also perpetuated by healthcare workers’ unexamined racist attitudes, beliefs, and biases when they interact with historically excluded populations within the system. The structural humility framework enables us to examine both institutionalized systems of power (macro-level structures) that limit vaccine access and the interpersonal communication (micro-level interactions) to capture the full spectrum of communication vulnerabilities experienced. The following section details the operationalization of structural humility within this study.

## Methodology

### Field Experiment Overview and Data

The audit test field experiment using three testers—an English-speaking female with a white name, an English-speaking female with a Latinx/e accent and Latinx/e name, and a Spanish-speaking female with a Latinx/e name—followed an IRB-approved script to inquire about COVID-19 vaccine registration and access via phone, email, and chatbot communications with SDH and MVS across all 50 U.S. states and the District of Columbia.^1^ Testers were instructed to inquire about ID requirements, health insurance, and vaccine procedures. When asked for identification, all testers offered a consular ID, which is common among some immigrant communities and has been politicized as an undocumented marker [67, 68]. Following inter-rater reliability tests to ensure consistent protocol use, data collection began in mid-June 2021. Testers made calls and sent emails during standard business hours and recorded details of each interaction, including wait times, disconnections, and non-responses. If no response was received after two weeks of follow up, interactions were marked as incomplete. In total, 456 out of 612 possible tester-organization interactions were completed; most failed connections occurred with MVS.

The quantitative analysis categorized the 456 interactions by types of interactions frequently experienced: translation service availability, residency and immigration status requirements, and negative connotations related to immigration status. The Spanish-speaking tester frequently faced abrupt call terminations due to lack of translation services and was less likely to receive clear vaccine information [69]. We further explored these findings in an in-depth analysis of the qualitative data to increase our understanding of the differences in communications and interactions experienced by the three audit testers. The qualitative data analysis sought to (1) identify systemic barriers that shape communication experiences, (2) understand how and during what points of the communication exchange vaccine access barriers emerged, (3) establish how the communication experience differed by language and racial profile of the tester, and (4) identify best communication strategies that address root causes of disparate healthcare inequities experienced by the Latinx/e/Spanish testers.

## Qualitative Data

### Instrumentation

The qualitative data collection tool designed to capture the tester communications in real time included the following topics: (1) communication medium (phone, email, or bot) and language utilized by the tester and SDH and MVS staff; (2) the length of time to connection with an individual or wait time for a response; (3) availability of translation support and languages offered; (4) information received on the COVID-19 vaccination, including where to receive it; (5) ID requirements; (6) availability of transportation support; (7) health insurance requirements; (8) other information provided about vaccine procedures, appointment reminders, and potential side effects; (9) the use of strategies for encouraging vaccine use; and (10) support for meeting documentation requirements. Each section of the form included a notes section to allow the tester to provide additional details and thick description of each interaction with SDH and MVS staff. The qualitative assessment of interactions included evaluation of the quality of translation services offered and (for the Spanish tester) received; extent of interaction; quality of information received; the nature of discussions regarding personal identification, residency requirements, transportation services, additional resources, and support; and the tone of SDH and MVS staff communications.

The qualitative data collection supported the interrogation of asymmetrical forms of power by considering first how the vaccine access infrastructure disenfranchises testers, which allowed us to situate micro-level interactions with SDH and MVS staff within the vaccine access infrastructure and its identified limitations. Importantly, our critical positionality via the structural humility framework does not view each point of contact or exchange with agency staff as the totality of the communication experience. Rather, structural humility positioned us to develop a qualitative data analysis process that would identify and evaluate every aspect of communication from the start of the engagement to the conclusion of the communication exchange. In other words, our qualitative data collection process captured each experience according to the organizational mechanism that permits race-based discrimination to occur within micro-level “street bureaucratic” discretion.

### Codebook

Guided by a structural humility orientation, a deductive and inductive codebook was developed to identify the spectrum of power dynamics embedded across the different forms of communication (phone, email, and chat), first from the experience of the Spanish speaking tester who was also the senior researcher and, thereafter, from the experience of the other testers. The deductive codes considered system-based barriers that prevented communication access, and inductive codes differentiated the type of discrimination or limited information accessed per interaction. To illustrate, deductive codes included error messages experienced at different communication entry points, non-translated operating systems, and the degree to which communication platforms recognized the Spanish language for Spanish language requests. This approach distinguished at what point in time during the vaccine access process different language users experienced prohibitive communication barriers and under what circumstances different language users had to navigate added barriers. For example, we distinguish an error message generated when the Spanish language speaker engaged with an English-only chat bot from an error message resulting from a bilingual chat-bot that had Spanish language functionality (and linked with a Spanish Question and Answer website page) but did not recognize the question submitted. While both experiences produced the same outcome—an inability to receive an answer to the question submitted—the bilingual chat-bot permitted engagement in native language and facilitated agency within the system.

Inductive and deductive codes were categorized into the following major groups: (1) dimensions of discrimination, (2) possible forms of discrimination, (3) exemplary communication strategies, and (4) Spanish-only communication barriers. The objective was to interrogate ethnocentric modes of communication, espoused power dynamics between the language user and agency staff, and the nuanced communication exchanges that intentionally and unintentionally reify racial and social hierarchies as communication occurs in real time. Per our theoretical orientation, we conceptualized “exemplary communication strategies” as specific examples of a healthcare agency staff espousing cultural competency, that is, best COVID-communication vaccine access practices and/or structural humility (shift in attitude) to address an experienced barrier that may prohibit vaccine access. Thus, the qualitative coding process allowed for differentiation when questions were not answered, when questions were defaulted to the provider, and what aspects related to identification and assumed identity were questioned or addressed during the communication exchange.

Thereafter, the first draft of the codebook was piloted three times utilizing a sample of 50 communication entries that differed in tester-language and communication platform utilized. The goal was to ensure the codebook comprehensively captured the full communication experience and that the spectrum of the interaction could be appropriately categorized and accounted for each time a user experienced system-based errors and/or communication barriers. To finalize the codebook, an additional 20 communication entries were piloted among two researchers. The interrater reliability was over 90%, which included the number of times each code was utilized within each communication entry.

Guided by the finalized codebook, the researchers engaged in an interactive process for achieving inter-rater consistency in qualitative coding [70], whereby each researcher was responsible for coding in a specific tester language, and a system was developed to address unitization and uncertainty in code applications. Specifically, researchers developed memos for codes they were uncertain applied to a unit of qualitative data, and bimonthly meetings between researchers were utilized to understand coding rationale, establish consensus of coding application, and resolve individual uncertainties. After completing coding of all communication entries, researchers reviewed codes for other entries (that they were originally not assigned) to identify any coding inconsistencies or clarifications and develop respective memos for regularly scheduled meetings. This process not only achieved across-and within-coder consistency but permitted a second level qualitative analysis described below.

### Analysis

All communication entries (*N* = 456) were uploaded into NVIVO for coding and analysis. Each communication record was uniformly organized by tester language, communication type, language utilized, medium of communication, and SDH vs. MVS staff interactions. The NVIVO qualitative survey analysis tool permitted qualitative queries to identify salient themes within and across categories, as well as the frequency of system-based barriers and types of communication barriers that contributed to disparate outcomes among the three language testers. In a second-level analysis, a researcher reviewed all responses that employed effective communication strategies and answered all questions regarding COVID-19 vaccine access to identify who among the language users had the agency to receive the COVID-19 vaccine based on the information provided. The overarching purpose of the second-level analysis was to move beyond understanding the totality of the communication exchange and understand the consequences of the communication exchange.

Below, we first present qualitative findings in Tables 1–6 that compare information across the three types of testers (Spanish-speaking, English speaker Spanish accent, and English-speaking). For each comparison in which the cell frequency is 3 or greater across the testers, we report a chi-square test that assesses the statistical significance of the differences between testers. We then detail in Table 7 the most salient experiences across language-testers and highlight exemplary communication strategies for building upon the structural humility shift in consciousness and addressing the centrality of racism embedded in the healthcare system (as observed in this study).

**Table 1:** Systemic barriers experienced per the tester’s language and racial profile

Systemic Barrier	Definition	Count		Spanish Speaker		English Speaker with Spanish Accent		English	
				F	%	F	%	F	%
English-Only Chat Bot	Chat function does not recognize Spanish language and/or no option to engage in the Spanish language	25		9	36%	7	28%	9	36%
				χ2=0.48 (*p*=0.787)					
Bilingual Bot Non-Functioning	Chat function supports communication in Spanish but the technology does not work (i.e., recognize questions, respond in Spanish, etc.)	2		2	100%	0	0	0	0
Address not Translated	Agency staff provides address of the vaccine location in English (i.e., utilizes English words for street number)	0		0	0	0	0	0	0
English-Only Phone System	Phone system does not include instructions for how to access translation (i.e., for Spanish press 2)	84		33	39%	35	42%	16	19%
				χ2=11.68 (*p*=0.003)					
Non-Spanish Ability	Agency staff only speaks English and is unable to secure a translator	20		20	100%	0	0	0	0
Non-Spanish Key Signal	Agency staff is able to secure a translator, but key communication is not translated in Spanish (i.e., please hold while I secure a translator)	22		17	77%	2	9%	3	14%
Translation Complete Via 3rd Party	Agency staff provides vaccine access information via a third party translator	34		34	100%	0	0	0	0
Translation Cost	Translator does not translate the information in its entirety; key information about vaccine access is (a) translated incorrectly or (b) not translated	27		27	100%	0	0	0	0
English-Only Form	Covid-19 vaccine access information platforms (e.g., inquiry form) are in English-only with no option to translate form questions or submission process	87		13	15%	40	46%	34	39%
				χ2=20.79 (*p*<0.00001)					
English-Only Email Response	Spanish language speaker submits a vaccine inquiry in Spanish and receives a response in English	8		8	100%	0	0	0	0
Limited Form Response	Automated information or email is in Spanish but the quality of the translation is poor and missing key information	7		6	86%	1	14%	0	0
Number and percent of total number of interactions evaluated		282	62%	135	92%	85	52%	62	43%
				χ2=87.86 (*p*<0.00001)					
		*N*=456		*N*=146		*N*=165		*N*=145	

## Findings

### Spanish-Speaking Tester Experiences

In illuminating the barriers to COVID-19 vaccine access and how they varied in frequency according to the racial and language profile of the tester, we begin with Table 1, which summarizes the frequency of systemic barriers experienced primarily, and sometimes only, by the Spanish-speaking tester. The most common barriers were associated with language access to the information, such as no instructions for how to access translation; the agency staff only speaks English and is unable to secure a translator; the agency staff secures a translator but key communication is not translated in Spanish; the translator does not translate the information in its entirety, and it is translated incorrectly or not translated at all; the chat function does not recognize the Spanish language and/or there is no option to engage in the Spanish language, and information platforms (e.g., inquiry form) are English-only and with no option for translation. Differences across the 11 systemic barriers experienced by the testers were statistically significant (*χ*^2^ = 87.86, *p* < 0.00001), with the Spanish language speaker most likely to experience them.

Table 2 illustrates how both overt racialized and judgmental language tones were primarily experienced by the Spanish-speaking speaker, and these differences were likewise statistically significant (*χ*^2^ = 82.35, *p* < 0.00001). These micro-level interactions with SDH and MVS staff also varied by the tester. When we analyzed the different tones of judgmental communication experienced among the testers, the Spanish tester experienced more judgmental language regarding their documentation status, whereas the English-speaking tester with a Spanish accent and English tester similarly experienced communication tones that were conclusive, annoyed, or hurried in nature to finish the communication exchange. Overt racialized language ranged from the agency staff choosing not to engage with the Spanish-speaking tester at all (i.e., hanging up the phone) or indicating in English an unwillingness to engage with the Spanish-speaking tester.

**Table 2:** Overt and covert language experience per the tester’s language and racial profile

Overt and Covert Language Experience	Description	Count		Spanish Speaker		English Speaker with Spanish Accent		English	
				F	%	F	%	F	%
Racialized language	Agency staff makes a negative insinuation when speaking with tester that is attributed to race, language, or immigration profile	18		18	100%	0	0	0	0
Judgmental Language	Agency staff communicates in negative manner or tone, including short, terse, frustrated, or exasperated; this includes written communication that does not invite additional questions and answers.	42		29	69%	5	12%	8	19%
				χ2=36.65 (*p*<0.00001)					
Number and percent of total number of interactions evaluated		60	13%	47	32%	5	3%	8	6%
				χ2=82.35 (*p*<0.00001)					
		* N*=456		* N*=146		* N*=165		* N*=145	

The dynamism of racialization is further illustrated in communication experiences regarding identification and identification status (see Table 3). The Spanish-speaking tester and English-speaking tester with a Spanish accent were more likely to have their identification politicized by the tester (*χ*2 = 22.61, *p* = 0.00001). Based on theorizing and emerging evidence on racialized administrative burdens [38, 71], our qualitative analysis suggests that these particular micro-level street bureaucratic discretions were racially motivated.

**Table 3:** Identification experience per the tester’s language and racial profile

Identification Experience	Description	Count	Spanish Speaker		English Speaker with Spanish Accent		English	
			F	%	F	%	F	%
Identification Questioned	Agency staff questions a person’s identification status	26	3	12%	13	50%	10	38%
			χ2=9.11 (*p*=0.010)					
Non-response to the identification	Agency staff does not answer questions related to identification	27	12	44%	7	26%	8	30%
			χ2=2.33 (*p*=0.311)					
Politicization of the Identification	Agency staff questions immigration status	28	19	68%	5	18%	4	14%
			χ2=22.61 (*p*=0.00001)					
Unknown Identification Status	Agency staff does not know if the consular identification will be accepted	7	3	42%	2	29%	2	29%
Unknown Identification Support	Agency staff is not familiar with the consular identification	1	0	0	0	0	1	100%
Residency Required	Agency staff communicates that residency status is required for vaccine access, including state-based residency	48	16	33%	20	42%	12	25%
			χ2=3.0 (*p*=0.223)					
Federal Identification Communicated	Federal identification policy communicated: (1) anyone can receive vaccine regardless of residency status; (2) no one can be turned away from vaccine receipt; (3) citizenship not required for vaccine access; (4) identification not required.	172	40	23%	82	48%	50	29%
			χ2=25.19 (*p*=0.00001)					
Consular identification not referenced	When listing possible forms of identification, the consular identification is not referenced.	10	3	30%	3	30%	4	40%
			χ2=0.3 (*p*=0.861)					
Identification Regulated	Identification is required for vaccine access and there are stipulations related to what kind of identification is accepted (i.e., photo, birthdate, etc.)	152	34	22%	70	46%	48	32%
			χ2=19.5 (*p*=0.00006)					
Identification Required	Identification is required for vaccine access	95	18	19%	41	43%	36	38%
			χ2=13.86 (*p*=0.0001)					
Number and percent of total number of interactions evaluated		566	148		243		175	
			χ2=38.1 (*p*<0.00001)					
		*N*=456	*N*=146		*N*=165		*N*=145	

When we accounted for all communication exchanges across testers, the SDH and MVS staff communicated the federal identification policy 37% of the time, although this, too, varied by the tester’s language and racial profile (see Table 4). Specifically, the English speaker and English speaker with a Spanish accent were told more often that no identification was required to receive the vaccine (*χ*^2^ = 29.92, *p* < 0.00001). In contrast, the Spanish-speaking tester was most likely to be first told that identification was needed or preferred (photo identification, birthdate, etc.), and after more communication regarding identification, to eventually receive communication that no identification was needed per the federal identification policy. This conversation dynamic illustrates how testers had disparate access to information. Regarding the latter, when we consider all parts of the federal policy, the Spanish tester received less information overall (*χ*^2^ = 31.23, *p* < 0.00001).

**Table 4:** Federal identification policy communicated per the tester’s language and racial profile

	Prohibitive Administrative Barriers Per Tester’s Language and Racial Profile	Count		Spanish Speaker		English Speaker with Spanish Accent		English	
				F	%	F	%	F	%
Federal Identification Communication Standard	A. Anyone can receive the vaccine and/or no one will be turned away	38		10	26%	25	66%	3	8%
				χ2=29.92 (*p*<0.00001)					
Federal Identification Communication Standard	B. No citizenship required for the vaccine (you do not have to be a citizen to receive the vaccine)	10		2	20%	3	30%	5	50%
Federal Identification Communication Standard	C. No proof of state residency required (you do not have to be a resident to receive the vaccine)	20		7	35%	6	30%	7	35%
				χ2=0.15 (*p*=0.928)					
Federal Identification Communication Standard	D. No ID is required to receive the vaccine	104		21	20%	48	46%	35	34%
				χ2=15.78 (*p*=0.00038)					
Federal Identification Communication Standard	A. Anyone can receive the vaccine, B. No citizenship required, C. No proof of state residency required, & D. No ID is required	2		0	0	0	0	2	100%
Federal Identification Communication Standard	B. No citizenship required, & D. No ID is required	2		0	0	1	50%	1	50%
Federal Identification Communication Standard	B. No citizenship required, C. No proof of state residency required	5		2	40%	1	20%	2	40%
Federal Identification Communication Standard	C. No proof of state residency required, & D. No ID is required	4		0	0	1	25%	3	75%
Federal Identification Communication Standard	A. Anyone can receive the vaccine, B. No citizenship required, C. No proof of state residency required	1		0	0	1	100%	0	0%
Federal Identification Communication Standard	A. Anyone can receive the vaccine & D. No ID is required	12		2	17%	9	75%	1	8%
Number and percent of total number of interactions evaluated		198	43%	44	30%	95	58%	59	41%
				χ2=31.23 (*p*<0.00001)					
		*N*=456		*N*=146		*N*=165		*N*=145	

Given that the Spanish-speaking tester disproportionately experienced systemic barriers, these findings highlight how language-based navigational agency can engender disparate healthcare equity outcomes within a healthcare system that reinforces English-only communications. The non-LEP individual (English-speaking tester) did not experience systemic barriers that prohibit access to life-saving information, whereas it was not unusual for the Spanish-speaking tester’s first experience with an English-only communication platform to be the conclusion of the communication exchange. In effect, LEP status prohibited the Spanish-speaking tester from engaging with the vaccine access infrastructure and hindered their ability to persist and receive potentially life-saving information.

### Administrative Barriers Experienced Across Testers

The most common vaccine access barriers encountered by the testers pertained to either (1) the agency staff asking the tester to engage in a process that required more of the tester (i.e., sending a vaccine locator as opposed to providing an address for vaccine receipt and requiring registration for the vaccine before providing nearest vaccine locations or answering any questions) or (2) not providing sufficient information (i.e., deferring questions to a specific vaccine provider, providing a phone number but not an address to the vaccine location, and/or not answering specific questions asked during the communication exchange). These “undue administrative barriers” were experienced by all of the testers, as shown in Table 5.

**Table 5:** Undue administrative barriers per the tester’s language and racial profile

Added Administrative Barriers Typology	Category	Number of times experienced by all Testers	Spanish Speaking Tester		English Speaker -Spanish Accent		English Speaking Tester	
			F	%	F	%	F	%
Asked for additional information that is not necessary to provide information access, vaccine registration, etc.	Added Adminis-trative Barrier	326	83	26%	133	41%	110	34%
			χ2=17.36 (*p*=0.00017)					
Added steps to find vaccine location (i.e., utilize link to find vaccine access map, navigate vaccine access locator, contact specific site for their location, etc.)	No address	208	62	30%	66	32%	80	38%
			χ2=3.87 (*p*=0.145)					
Agency staff does not know the answer to the question and concludes the communication without providing information access to inquiry	Questioned not Answered	198	83	42%	80	40%	35	18%
			χ2=35.80 (*p*<0.00001)					
Agency staff includes partial response to the question and concludes the communication without providing complete information	Absence of Additional Information	95	27	28%	39	41%	29	31%
			χ2=3.92 (*p*=0.141)					
Agency staff responds to question by defaulting the question to the specific vaccine provider; the communication is concluded without providing complete information	Defaulted to Provider	187	46	25%	45	24%	96	51%
			χ2=40.93 (*p*<0.00001)					
Instead of providing an address based on the person’s location, agency staff directs the person to a general location (i.e., nearest CVS or Walmart)	General Location	12	7	58%	4	33%	1	9%
Email, home address. and/or phone number needed in order to access vaccine information and vaccine registration processes	Prohibitive Adminis-trative Barrier	19	3	16%	1	5%	15	79%
Automated response indicates a follow-up communication will be received, but there is no follow-up	Non-Engagement	22	7	32%	11	50%	4	18%
			χ2=5.05 (*p*=0.080)					
Automated information is not up to date with vaccine access information (i.e., prioritizing groups for vaccine access post the stated timeline)	Wrong Information	49	20	41%	18	37%	11	22%
			χ2=4.10 (*p*=0.129)					
Resources communicated (i.e., Frequently Asked Question document, text-based reminder, website link with information about registration access) are not sent and/or received	Resources not Sent or Received	6	2	33%	1	17%	3	50%
Number and percent of total number of interactions evaluated		1122	340		398		384	
			χ2=7.35 (*p*=0.025)					
		*N*=456	*N*=146		*N*=165		*N*=145	

Table 5 shows the high total frequency of administrative barriers experienced (*n* = 1,122) and that the English-speaking tester was more likely to be asked to provide additional information or engage further with the vaccine information infrastructure to receive information. However, analysis of the typology of undue administrative barriers by language and race showed that the English speaker with a Spanish accent experienced a higher frequency of not being provided an address and of questions being deferred to a provider. The Spanish-speaking tester experienced a higher frequency of questions not being answered and receiving incorrect information.

To further unpack the nuance of undue administrative barriers, we distinguished experiences that we believe were vaccine access prohibitive in nature, that is, those that not only caused the tester to engage in additional interactions, but that also heightened the risk of the tester being pushed out of vaccine access. Table 6 shows that the Spanish language speaker experienced more technical issues, often language-based, that terminated the communication (*χ*^2^ = 37.38, *p* < 0.00001) and prohibited vaccine information access, which we refer to as a *racialized administrative burden*, for two reasons. First, the imposition of administrative burdens and language barriers presents an amplified obstacle to accessing essential public benefits, and second, coupling prohibitive administrative barriers and racialized administrative burdens exacerbates the range of oppression and discrimination in the vaccine infrastructure.

**Table 6:** Prohibitive administrative barriers per tester’s language and racial profile

Prohibitive Administrative Barriers	Definition	Number of times experienced by all Testers	Spanish Speaking Tester		English Speaker -Spanish Accent		English Speaking Tester	
			F	%	F	%	F	%
Technical Issues	Technical issues that prohibit access to information, established connection to service provider, and/or end communication and prohibit any information access	321	148	46%	78	24%	95	30%
			χ2=37.38 (*p*<0.00001)					
Prohibitive Information Requested	Agency staff needs a home address to register the person for the vaccine and/or provide information about the vaccine. There is no information provided without a home address and completion of the registration process.	368	93	25%	111	30%	164	45%
			χ2=33.32 (*p*<0.00001)					
Wrong Information Provided	Agency staff provides wrong information (i.e., associated cost that the person will have to assume, residency requirements) that may be experienced as prohibitive to the person seeking to access the vaccine	407	122	30%	127	31%	158	39%
			χ2=8.41 (*p*=0.015)					
Insurance Card or Fee Required	Agency staff communicates that a person needs to have insurance to receive the vaccine	36	7	20%	13	36%	16	44%
			χ2=5.25 (*p*=0.072)					
Number and percent of total number of interactions evaluated		1132	370		329		433	
			χ2=21.82 (*p*=0.00002)					
		*N*=456	*N*=146		*N*=165		*N*=145	

### Exemplary Communication Strategies and Systemic Barriers

Our analysis revealed that SDH and MVS staff at various points in communication employed culturally competent communication, promoted vaccine access, and demonstrated structural humility. Examples include nudges throughout the conversation to encourage signing up for a vaccine appointment at different points during the communication exchange; going beyond basic translation services to augment the information that was available; and, at times, utilizing cultural references to encourage vaccine use. All testers, regardless of language or racial profile, encountered some positive interactions. However, even when exemplary communication strategies were used, systemic barriers such as English-only phone systems or translation errors still led to incomplete or incorrect information. Testers often did not receive answers to all of their questions or were given incorrect details about federal ID requirements or vaccine site locations.

To overcome these barriers, we recommend policy and practice changes that target the root causes of healthcare disparities (Table 7). For example, we recommend that agency staff equip LEP populations with navigational agency through access to additional information and resources and authentic choices that support equitable healthcare access. Moreover, agencies need to assume a modality of work that allows for added labor, time, and teamwork that is required to provide effective translation and support for historically excluded populations. We also outline the structural humility framework shift^2^ necessary to recognize and address the centrality of racism embedded within the vaccine delivery system.

**Table 7:** Policy and praxis recommendations for healthcare equity

Communication platform	Policy to praxis recommendation	Structural humility framework shift
Phone	Bilingual agency staff need to have fluent, native-level speaking abilities in the language they translate to English in order to understand all components of the conversation, effectively employ best communication practices within cultural nuance, as well as understand additional language-barriers pertaining to healthcare access that LEP populations experience post phone inquiry for healthcare access. Should a non-bilingual agency staff need translation support services to provide fluent-level language support, the non-bilingual agency staff needs to be able to communicate key phrases in the native language of choice (i.e., “please hold while I secure a translator”) either by the agency staff or through a pre-recorded message	LEP populations interface with the consequences of English-only laws, policies, and practices when in fact the United States did not have a national language for the supermajority of its existence. These dynamics may be perpetuated at an interpersonal level per agency staff bias and/or at a local, statewide and federal level per the sociopolitical histories that shape corresponding policies and practices (i.e., number of anti-immigrant laws in an immigrant restrictive state). Healthcare access necessitates agency staff understand how English-only laws, policies, and practices prohibit equitable access; consequently, a fluent and native speaking standard for bilingual agency staff interrogates inequitable healthcare access environments
Phone	Upon learning about external factors that prohibit healthcare access, agency staff need to assume an advocacy role to facilitate additional administrative barriers encountered by LEP populations and call attention to the added language support to facilitate equitable healthcare access. When espousing an advocacy role, agency staff need to reiterate that the communication between themselves and LEP person is an open pathway that can be accessed post interaction should additional support be needed	The process of accessing healthcare services is not a one-point in time interaction; rather, until the person receives their needed healthcare service, the LEP will be susceptible to English-only laws, policies, and practices at a personal, local, state and federal level
Phone	Agency staff need to be attuned to how anti-immigrant laws at federal and state levels implicate healthcare access policy and praxis to communicate effective strategies that keep immigrants and LEP populations safe while they access healthcare services	Anti-immigrant rhetoric, policy, and practice are compounded forms of LEP marginalization irrespective if the LEP person who is seeking healthcare services experiences anti-immigrant barriers that heighten healthcare inequity. Subsequently, facilitating healthcare access while addressing potential anti-immigrant barriers is healthcare equity
Phone	Agency staff need to make their navigational agency known to LEP populations. By extension, LEP populations should have access to additional resources, and services provided for healthcare access. When agency staff equip LEP populations with navigational agency, they advance authentic choices among LEP populations and realize healthcare equity access	Information access is navigational capital and agency; agency staff are on the frontlines of reducing barriers that prohibit navigational agency and capital. Subsequently, information access is a component healthcare equity access
Phone	Successfully securing a translator to mediate healthcare access requires additional labor, time, and requires teamwork between the non-bilingual agency staff and the translator. Consequently, agency staff should assume a modality of work that permits added labor, time, and teamwork to translate, as translation oftentimes requires additional more explanation on behalf of the translator for the cross-language communication to be effective	Providing translation services is not an added cost to the healthcare system. Rather in order for healthcare equity access to be realized, communicating across different languages within a multilingual country is the assumed work norm for healthcare organizations to realize their vision and mission, as well as ensure the healthcare as a public good
Email/form	If a response necessitates additional administrative barriers to receive healthcare access, processes and strategies for access must be communicated in a manner that LEP populations will understand	To bridge the digital divide and disparate reading levels that compound marginalization for historically excluded populations, written communication and educational resources need to employ popular education techniques to demystify healthcare access processes
Email/form	Upon responding to inquiry, agency staff need to provide all related, additional information so LEP populations have the context to understand next steps in processes and additional support available to them	E-mail communication is an opportunity to learn more about barriers that prohibit healthcare access; therefore, agency staff need to keep lines of communication open and regard this communication as an opportunity to provide additional support post email communication should LEP populations experience additional healthcare challenges
Email/form	Where possible, agency staff communicate in a tone that encourages the lines of communication to remain open, as well as reassuring LEP populations that clarifying questions and request for support is welcomed	Email communication is an opportunity to personalize healthcare access and combat communication automation that does not regard the unique healthcare access barriers that LEP populations experience and compound marginalization
Email/form	Response to email inquiries need to bridge frequently asked questions and strategies with the specific concerns raised by LEP populations	Agency staff should not assume that people seeking support services are familiar with healthcare access procedures, email communication should seek to educate as much as clarify resolution to their inquiry as well as proactively address experienced additional vulnerabilities due to their LEP status
Email/form	Agency staff need to be aware of when prohibitive policies and practices may interfere with healthcare access to effectively communicate strategies that ensure healthcare services and protect the safety of historically excluded populations	Agency staff have navigational agency to bypass prohibitive healthcare access barriers. Should their respective healthcare organization have prohibitive policies and practices, agency staff have an ethical obligation to communicate how else LEP populations can receive healthcare access
Email/form	When possible, agency staff need to invite and inform people seeking healthcare services about incentive programs and community support services. This communication needs to include resources that make the participation of historically excluded populations possible	Healthcare access are opportunities for people to be in community with one another. The ability to address the needs of marginalized populations while being inclusive in the co-construction of community addresses previously experienced disenfranchisement within healthcare access
Communication Infrastructure System	The phone operating system allows LEP populations to recognize translation support within the phone-operating system. There is parity between the information provided to fluent English speakers and LEP populations per the phone operating system	Providing translation services should not be limited to the process of translating verbal communication; rather, the need for translation services begins at the first-step of seeking healthcare services across various information-seeking pathways (i.e., phone, email, healthcare website, and chat function)
Communication Infrastructure System	Communication operating system needs to support multilingual utility and function, as need for language support varies per the needs of LEP populations in the United States	Bilingual support services should not be mistaken for multilingual support services provided. Healthcare organizations need to understand in-depth the diverse language needs of the populations they serve and which historically excluded populations have the least language access to ensure healthcare equity
Communication Infrastructure System	Communication systems are oftentimes the first line of contact with historically excluded populations, including LEP populations. Thus, communication systems should make all resources, services, and information access available, so LEP have information access equity and can follow-up with an agency staff should they have outstanding needs, questions, and/or support. An informed, multilingual phone operating system offloads agency staff labor, establishes support and care provided for LEP, and cultivates a helpful approach among agency staff	First-line of contact establishes the extent to which historically excluded and LEP populations are seen and their needs understood. Communication platform systems are an extension of healthcare access equity and should be regarded as tools that can address prohibitive administrative barriers
Communication Infrastructure System	Automated follow-up communication are opportunities to reiterate significant information central to healthcare access, empower historically excluded populations with strategies to combat various forms of discrimination, as well as address ongoing forms of vulnerability experienced by historically excluded populations and LEP populations	Automated follow-up communication may be the only source of official information historically excluded populations and LEP populations. Thus, automated communication needs to establish an inclusive and accessible standard to advance healthcare access and literacy

## Discussion

Our critical methodology and direct engagement with macro-level communication infrastructure and micro-level interactions allowed us to excavate multidimensional forms of marginalization among Latinx/e Spanish speakers, who comprise the majority of the LEP population. While Latinx/e Spanish speakers do not represent a single racial group, those perceived to be of Latinx/e origin and who speak Spanish experience a racialization process shaped by dominant U.S. social structures—one that perpetuates their marginalization within and beyond the healthcare system. Although we cannot conclude whether individuals speaking different languages or presenting as other racial or ethnic groups would have encountered the same negative outcomes, the racialized and hierarchical nature of healthcare infrastructure suggests that the absence of translation and culturally responsive communication also disadvantages other LEP populations. When we examined variation in patterns of barriers and marginalization experienced by regions and states with higher vs. lower LEP populations, we did not find statistically significant differences; that is, they are widespread. Therefore, improving policies, systems, and processes for Spanish-speaking LEP communities represents not only a corrective action for disproportionately affected groups but also a necessary shift in consciousness toward equitable outcomes for all LEP individuals.

The forms of marginalization and disparate treatment detailed in our findings oftentimes remain invisible to healthcare scholars, policymakers, and even well-meaning healthcare agency staff. Our research revealed how healthcare disparities persist, in part, because unexamined forms of racism within the healthcare infrastructure remain embedded within the infrastructure of service delivery and are expressed through routine practices. These macro-level systems and micro-level interactions are frequently regarded as business-as-usual approaches to attending to the needs of LEP populations. For instance, a monolingual English speaker may hear “For Spanish press two” and conclude that translation services are indeed being offered without thinking, “Can a Spanish speaking person understand the English phrase that references how to access translation?” or “Is there bilingual staff available to support an LEP person in accessing lifesaving information after pressing two?” And perhaps most importantly, why does a healthcare communication platform system have disparate levels of information available for non-native English speakers, and why are we not making this information available in people’s native languages? To arrive at the latter from the experience of the LEP populations, this study facilitates an understanding of how DHS and MVS are inherently racialized organizations whose policies and practices have been co-constructed by systemic inequality; [72, 73] whiteness is indeed a credential that facilitates access to lifesaving information and vaccines.

Policies and practices that promote navigational agency for LEP individuals (detailed in Table 7) hence need to be accompanied by system-level reforms to address root causes of inequality. Our research demonstrates how disparate race-based healthcare services remain embedded in present-day organizational schemas, dispelling the notion that healthcare inequities only emerge when bias is overt or intentional. Distinguishing between implicit and structural discrimination deepens our understanding of how racialized organizations co-produce disparate race-based outcomes within healthcare settings in the context of seemingly race-neutral policies and practices. We accordingly emphasize the importance of structural humility not just as an established framework shift for understanding the various facets of marginalization that LEP populations experience, but also as a tool that actively resists discrimination while elevating the needs and experiences of LEP populations.

This study contributes to the field in multiple ways. First, by centering the experiences of the most vulnerable tester (Spanish speaker), we conscientiously align our analytical approach with the populations most directly affected by vaccine access inequities, immigrant, and undocumented communities. In addition, in critically examining the extent to which the Spanish language tester was disenfranchised from the vaccine access healthcare system and how unexamined racist attitudes, beliefs, and biases arose during the communications, the qualitative data analysis permitted a more nuanced, in-depth understanding of the dynamism of hierarchical forms of power that perpetuate disenfranchisement across language and accent. And methodologically, our research demonstrates how to assess the praxis of structural humility—i.e., grounding the qualitative data analysis methodology and presupposing the sociopolitical context that immigrant and undocumented populations navigate to access the COVID-19 vaccine—to advance justice research methods and the development of equity-centered policies and practices.

We also acknowledge a key limitation of our study that examined individual interactions around vaccine access at a specific point in time and for a specific ethnic/racial subgroup. Since the time of our data collection, the U.S. healthcare and political landscape has continually shifted. For instance, we are experiencing in real time the consequences of a new presidential administration that further racializes people of Latinx/e origin. The recent Executive Order that designates English as the official language of the U.S. [74] targets non-English speakers and restricts access to critical services, likely exacerbating barriers documented in our study, and not only for those of Latinx/e origin. Achieving healthcare equity in policy and praxis will require a sustained, coordinated, and intersectional effort to address root causes of inequality that began in earnest when the U.S. government coordinated with healthcare organizations on a nationwide scale to address disparate race-based access to healthcare services, and by extension, engaged with healthcare language rights as well. Much of the work that remains is being rolled back politically, likely to the detriment of our collective wellbeing that is bound together with equity for LEP populations.

## Conclusion

The objective of this study was to evaluate how state health agencies communicated vaccine requirements and whether that varied based on the perceived race and language of the individual seeking information. Our empirical analyses confirmed significant variation across states and individual demographic and language profiles. Most notably, the Spanish-speaking individual most faced communication barriers, received less information, and experienced racialized and linguistic discrimination that impeded vaccine access. This study presents best policies and practices that build upon culturally competent strategies vis-à-vis structural humility to support an improved healthcare equity infrastructure at macro and micro levels, safeguarding Spanish speakers from unintentional forms of bias and discrimination.

## Data Availability

The data supporting the findings of this study has been submitted per the publishing requirements.

## Footnotes

1 Testers were female only because of research resource constraints, but also given prior research [68] showing that 67 percent of COVID information hotline callers were female.

2 Framework shifting is a public policy concept whereby research findings are utilized to transform. how people think about societal issues and policy choices [74]. Structural humility is operationalization of an equity in healthcare framework shift see [75].
